# A novel score system based on arginine metabolism-related genes to predict prognosis, characterize immune microenvironment, and forecast response to immunotherapy in IDH-wildtype glioblastoma

**DOI:** 10.3389/fphar.2023.1145828

**Published:** 2023-04-05

**Authors:** Wentao Feng, Mingrong Zuo, Wenhao Li, Siliang Chen, Zhihao Wang, Yunbo Yuan, Yuan Yang, Yanhui Liu

**Affiliations:** Department of Neurosurgery, West China Hospital of Sichuan University, Chengdu, Sichuan Province, China

**Keywords:** arginine, glioblastoma, IDH-wildtype, metabolism, prognosis, tumor microenvironment, immune infiltration, immune checkpoint inhibitor

## Abstract

**Introduction:** Glioblastoma is one of the most lethal cancers and leads to more than 200,000 deaths annually. However, despite lots of researchers devoted to exploring novel treatment regime, most of these attempts eventually failed to improve the overall survival of glioblastoma patients in near 20 years. Immunotherapy is an emerging therapy for cancers and have succeeded in many cancers. But most of its application in glioblastoma have been proved with no improvement in overall survival, which may result from the unique immune microenvironment of glioblastoma. Arginine is amino acid and is involved in many physiological processes. Many studies have suggested that arginine and its metabolism can regulate malignancy of multiple cancers and influence the formation of tumor immune microenvironment. However, there is hardly study focusing on the role of arginine metabolism in glioblastoma.

**Methods:** In this research, based on mRNA sequencing data of 560 IDH-wildtype glioblastoma patients from three public cohorts and one our own cohort, we aimed to construct an arginine metabolism-related genes signature (ArMRS) based on four essential arginine metabolism-related genes (ArMGs) that we filtered from all genes with potential relation with arginine metabolism. Subsequently, the glioblastoma patients were classified into ArMRS high-risk and low-risk groups according to calculated optimal cut-off values of ArMRS in these four cohorts.

**Results:** Further validation demonstrated that the ArMRS was an independent prognostic factor and displayed fine efficacy in prediction of glioblastoma patients’ prognosis. Moreover, analyses of tumor immune microenvironment revealed that higher ArMRS was correlated with more immune infiltration and relatively “hot” immunological phenotype. We also demonstrated that ArMRS was positively correlated with the expression of multiple immunotherapy targets, including PD1 and B7-H3. Additionally, the glioblastomas in the ArMRS high-risk group would present with more cytotoxic T cells (CTLs) infiltration and better predicted response to immune checkpoint inhibitors (ICIs).

**Discussion:** In conclusion, our study constructed a novel score system based on arginine metabolism, ArMRS, which presented with good efficacy in prognosis prediction and strong potential to predict unique immunological features, resistance to immunotherapy, and guide the application of immunotherapy in IDH-wild type glioblastoma.

## Introduction

Glioblastoma, a type of malignant diffuse glioma, presented with extremely poor prognosis (Stupp et al., 2005). Despite after standard treatment regime, including surgery, chemotherapy, and radiotherapy, the prognosis of IDH-wildtype glioblastoma patients remains unsatisfactory with a median overall survival of fewer than 2 years (Chinot et al., 2014; Gilbert et al., 2014; Stupp et al., 2015). Therefore, many studies aimed to explore brand-new therapies to improve overall survival of glioblastoma patients. Abundant attempts of multiple novel treatments have failed to improve overall survival of glioblastoma patients (Westphal et al., 2013; Chinot et al., 2014; Gilbert et al., 2014; Wick et al., 2017). Immunotherapy, which aims to discharge the immunosuppressive microenvironment of tumors and enhance anti-tumor effects delivered by immune cells, has been proved effective in multiple cancers, including non-small-cell lung cancer (Janjigian et al., 2021; Kamdar et al., 2022), melanoma (Reck et al., 2016), breast cancer (Larkin et al., 2015), and digestive tract cancer (Larkin et al., 2015). However, most of the attempts for the application in immunotherapy in glioblastoma eventually failed to improve overall survival (Reardon et al., 2020; Lim et al., 2022; Omuro et al., 2022). The reasons of these failure for the application of immunotherapy in glioblastoma have attracted a lot of attention and researchers. The unique immunological behavior and environment of brain was considered as a critical reason. However, some studies which aimed to apply immune checkpoint inhibitors (ICIs) in metastatic brain tumors have succeeded to improve patients’ overall survival (Tawbi et al., 2018; Hendriks et al., 2019), which suggested that the unique immune microenvironment of brain may not be the key reason for the failures in glioblastoma. Other studies also attempted to alter the timing of immunotherapy use. Inspiringly, the application of neoadjuvant ICIs in glioblastoma can enhance the immune response and convert immunological features (Cloughesy et al., 2019; Schalper et al., 2019). These studies encouraged us that if we can get a better understand of immunological behaviors of glioblastoma, we may be able to reshape immunological features and ease the resistance to immunotherapy in glioblastoma.

Metabolism pattern of cancer cells and its impact on the immunological features of tumor microenvironment is becoming more and more attractive to researchers. Based on public database and our own data, we have explored the correlation of several compounds and amino acid metabolism with the immune microenvironment of glioma (Chen et al., 2022a; Chen et al., 2022b; Zhang et al., 2022). However, formation of the unique immunological features of glioma is an extremely complex process and is influenced by the metabolism pathways of massive compounds in cancer cells. None of any separated metabolism pathway can totally explain the special immune microenvironment of glioma. Hence, here we intended to explore the relationship between tumor immunological features and another conditionally essential amino acid for human, arginine, which functions as a precursor for synthesis of multiple compounds, including urea, nitric oxide, proline, glutamate, creatine, and agmatine (Morris, 2006) and plays key roles in cell growth and survival (Delage et al., 2010). Most cancer cells lose the capacity of intracellular arginine synthesis because of the loss of a key enzyme that produces arginine, argininosuccinate synthetase 1 (ASS1) (Bronte and Zanovello, 2005). Consequently, cancer cells depend on exogenous arginine to meet the demands, indicating a unique pattern of arginine metabolism in cancers. Besides, arginine was also proved to execute powerful regulation for immune system (Dillon et al., 2004; Wheatley et al., 2005). Arginine could modulate T Cells metabolism and enhance their anti-tumor activity (Geiger et al., 2016). The cell cycle of T cells was also influenced by arginine availability (Rodriguez et al., 2007). The large consume of extracellular arginine by cancer cells decreases the arginine level in the tumor microenvironment (TME). Moreover, tumor-associated macrophages (TAMs) could produce arginase, an enzyme to degrade arginine, to decrease the arginine level in TME (Currie, 1978; Currie et al., 1979). These synergistic effects contribute to shortage of arginine in TME and lead to dysfunctions of T cells, resulting in an immunosuppressive phenotype. Therefore, to prevent arginine degradation and replenish arginine supply in the TME can enhance the anti-tumor effects delivered by T cell and NK cell (Geiger et al., 2016). These studies indicated that arginine plays an essential role in tumor progression and process of antitumor immunity, and the metabolism of arginine has a significant impact on immunological feature of tumors. However, the role of arginine metabolism in the progression and immune landscape of glioblastoma was still not well elucidated.

Different with our previous studies, to avoid potential disturbance from tumor heterogeneity, we tried to focus on a subdivision of glioma, IDH-wildtype glioblastoma, which may result in smaller cohort scale but more convinced evidence. In this study, we included multiple IDH-wildtype glioblastoma patients’ cohort, including TCGA, CGGA325, CGGA693, and our own cohort, to explore how the unique arginine metabolism pattern of cancers influenced the malignant behaviors and immunological features in glioblastoma. We filtered all genes that were related to arginine metabolism and found out essential genes that had most influences on glioblastoma. Then, based on these essential genes, we constructed a score system, which was named as arginine metabolism-related gene signature (ArMRS) and showed with its satisfactory efficiency on prognosis prediction. Furthermore, we conducted multiple analyses to elucidate the relationship between ArMRS and immunological features of glioblastoma. Additionally, we also put forward the potential ability of ArMRS to predict response to immunotherapy. Based on these analyses, we hope to explore the potential applications of arginine metabolism in improving responses to immune checkpoint inhibitors and guiding selection of immunotherapy in IDH-wildtype glioblastoma patients. Combination of this study and our previous studies may contribute to establishing a more detailed model that comprehensively explained the relationship between metabolism and immune of glioma.

## Materials and methods

### Patient cohorts and data preprocessing

Gene expression profiles (fragments per kilobase million, FPKM) and clinicopathological features in this research were fetched from three public datasets and extracted from the mRNA-seq data of our own patient cohort. Those patients diagnosed with primary IDH wild-type glioblastoma were included in this research. Those patients with recurrent glioblastomas or under 18 years old were excluded from this research. The three public cohorts consisted of one cohort from the Cancer Genome Atlas (TCGA, https://portal.gdc.cancer.gov/) and two cohorts from the Chinese Glioma Genome Atlas (CGGA, http://www.cgga.org.cn/). The cohort from TCGA contained 237 primary IDH-wildtype glioblastoma samples and 234 of which had complete survival data. The two cohorts from CGGA were CGGA325 and CGGA 693 cohorts, which contained 112 and 175 primary IDH-wildtype glioblastoma samples, respectively.

Our own cohort contained 36 primary IDH-wildtype glioblastoma patients from West China Hospital (WCH). We gained these tumor samples during resection surgery and then sequenced them for mRNA. Subsequently, the mRNA sequencing data was quantified and normalized to FPKM using STAR. The survival data of these 36 patients was recorded through regular follow-up every 3 months. Besides, the genes with too low FPKM values (maximum FPKM < 0.1 or standard deviation < 0.01) were excluded from further analyses in succeeding preprocessing procedure. Table 1 listed out detailed clinicopathological features of the patients in all these four cohorts.

**TABLE 1:** Clinicopathological characteristics of patients in TCGA, CGGA325, CGGA693, and WCH cohorts.

Characteristics	TCGA (N = 237)	CGGA325 (N = 112)	CGGA693 (N = 175)	WCH (N = 36)
Age: mean(range)	60 (21–89)	51 (18–79)	50 (19–76)	52 (19–77)
Gender				
Female	93 (39.2%)	39 (34.8%)	79 (45.1%)	11 (30.6%)
Male	143 (60.3%)	73 (65.2%)	96 (54.9%)	25 (69.4%)
NA	1 (0.4%)	0	0	0
Histology				
Glioblastoma	237 (100%)	112 (100%)	175 (100%)	36 (100%)
Grade				
G4	237 (100%)	112 (100%)	175 (100%)	36 (100%)
IDH status				
Wild-type	237 (100%)	112 (100%)	175 (100%)	36 (100%)
TERT promoter status				
Mutant	167 (70.5%)	NA	NA	10 (27.8%)
WT	17 (7.2%)	NA	NA	19 (52.8%)
NA	53 (22.4%)	NA	NA	7 (19.4%)
MGMT promoter status				
Methylated	129 (54.4%)	73 (65.2%)	67 (38.3%)	12 (33.3%)
Unmethylated	78 (32.9%)	35 (31.3%)	79 (45.1%)	14 (38.9%)
NA	30 (12.7%)	4 (3.6%)	29 (16.6%)	7 (19.4%)
ATRX status				
Mutant	8 (3.4%)	NA	NA	28 (77.8%)
WT	225 (94.9%)	NA	NA	7 (19.4%)
NA	4 (1.7%)	NA	NA	1 (2.8%)

Abbreviation: TCGA, the cancer genome atlas; CGGA, chinese glioma genome atlas; WCH, west china hospital; IDH, isocitrate dehydrogenase; TERT, telomerase reverse transcriptase; MGMT, O6-methylguanine-DNA, methyltransferase; ATRX, alpha-thalassemia x-linked intellectual disability syndrome; WT, wild type; NA, not available.

### Definition of essential arginine metabolism-related genes and construction of the arginine metabolism-related genes risk signature

The arginine metabolism-related genes (ArMGs) were exported from the Molecular Signature Database (MSigDB) with the keyword “arginine metabolic process”, “arginine transport”, “arginine catabolic process”, “arginine biosynthetic process”, and 26 genes were kept after excluding lowly expressed genes. After that, we constructed a gene risk signature based on the expression levels of several essential ArMGs to explore the relationship between arginine metabolism and the malignancy of glioblastoma, which was named as arginine metabolism-related genes risk signature (ArMRS). First, we split the TCGA cohort into training and validation sets with a ratio of 6:4. The other three cohorts were utilized as validation cohorts. The 26 ArMGs were filtered using the Least Absolute Shrinkage and Selection Operator (LASSO) Cox regression analysis in the training set. If the coefficient of an ArMG was not zero at the optimal model with maximum C-indices in over 100 random repetitions of LASSO Cox regression, this ArMG was defined as an essential ArMG in glioblastoma. Subsequently, we fitted a concluding multivariate Cox regression model to the training set with the essential ArMGs. The ArMRS was calculated with the following formula:

$$ArMRS=\underset{i=1}{\sum }\left({\beta_{i}*\mathrm{Exp}}_{i}\right)$$

In this formula, the *β* and *Exp* represented the coefficients and expression levels of each essential ArMG in the final multivariate Cox regression, respectively. Moreover, we determined the optimal cut-off value of ArMRS in each cohort by using the “surv_cutpoint” function in the R package “survminer” with group proportion ≥ 0.3. Based on these cut-off values, all patients of these four cohorts were classified into ArMRS high-risk group or low-risk group. Eventually, we illustrated the receiver operating characteristic (ROC) curves in validation sets of 6-, 12-, and 18-month survival rates and used the R package “time ROC” to calculate the area under the ROC curve (AUC) to validate the efficacy of the prognostic prediction.

### Functional enrichment analyses based on ArMRS risk groups

R package “limma” was utilized to identify differentially expressed genes (DEGs) between ArMRS risk groups. Those genes with adjusted *p*-value <0.05 and |log_2_FC| > 0.5 were defined as DEGs. To perform gene set enrichment analyses, Gene set enrichment analysis (GSEA) and over-representation were used to evaluate the differentially expressed genes (DEG) with Gene Ontology (GO) enrichment using the R package “clusterProfiler” based on different ArMRS risk groups. Moreover, we transferred the logFPKM matrix of genes to the pathway expression matrix using the R package “GSVA” and used the “limma” package to identify the differentially expressed pathways between risk groups.

### Analyses of gene alterations and copy number variation

We obtained the gene alterations and copy number variations (CNV) data of patients of the TCGA cohort from the cBioPortal database (https://www.cbioportal.org/) to elucidate the different patterns of gene alterations and CNVs between different ArMRS risk groups. The R package “maftools” was used to illustrate the gene alterations. Besides, the mean Genomic Identification of Significant Targets in Cancer (GISTIC) score of 1 Mb chromosome segments was used to depict the CNV levels.

### Nomogram construction based on ArMRS and other potential prognostic factors

To construct a nomogram based on ArMRS that could effectively predict glioblastoma patients’ prognosis, we utilized the univariate and multivariate Cox regression analyses to clarify independent prognostic factors. Firstly, the ArMRS, together with other potential prognostic patient and tumor factors, including age, gender, KPS, MGMT promoter methylation status and TERT promoter mutation status, were included in the univariate Cox regression analysis. Subsequently, those prognostic factors with *p*-value < 0.05 in the univariate Cox regression analysis were enrolled into the following multivariate analysis. Those factors with a *p*-value <0.05 in multivariate Cox regression analysis were defined as independent prognostic factors.

The nomograms were also constructed based on the prognostic patient factors with *p*-value < 0.05 in the univariate Cox regression analysis as well as the adjuvant therapies, using the R package “rms”. To evaluate the efficacy of nomograms in the prediction of prognosis, we computed calibration curves for each nomogram.

### Analyses of the association between ArMRS and immunological features, and prediction of response to immunotherapy in glioblastoma

To elucidate the impact of arginine metabolism on the tumor immune microenvironment, we performed multiple analyses to characterize the differences in the tumor immune microenvironment between different ArMRS risk groups. First, we utilized the web-based CIBERSORTx suite (https://cibersortx.stanford.edu/) to compute the absolute infiltration fraction of 22 types of immune cells in glioblastoma based on the LM22 reference gene signature. Subsequently, the immune microenvironment-related scores, including stromal and immune scores, were evaluated by a previously reported algorithm, the Estimation of Stromal and Immune cells in Malignant Tumor tissues using Expression data (ESTIMATE) (Yoshihara et al., 2013). Additionally, we also evaluated the tumor purity according to the ESTIMATE score and consensus purity estimation (CPE) data published by Aran et al. (2015). To assess the tumor immunological phenotype (TIP), we utilized another previously published algorithm (Wang et al., 2021) to calculate the TIP gene signature. According to the TIP gene signature, we could determine the immunological phenotype of tumor as either relatively “cold” or “hot” tumors. Finally, the TIDE suite (https://tide.dfci.harvard.edu/) was utilized to perform *in silico* analyses to predict the response to immune checkpoint inhibitors therapy in glioblastomas.

### Statistical analysis

We used the R software (version 4.2.1) to conduct all the above bioinformatic analyses unless otherwise specified. We used the Wilcoxon rank sum test to evaluate the differences between different ArMRS risk groups for continuous variables. The chi-square test was used to evaluate the differences for categorical variables. All the survival analyses were conducted using the R package “survminer”. The log-rank test was utilized to test the differences between Kaplan-Meier (K-M) curves. The “coxph” function of the R package “survival” was used to conduct univariate and multivariate Cox regression analyses. The LASSO Cox regression analysis was performed using the R package “glmnet”. The T Iterative Grubbs test was utilized to exclude the outliers in linear regression analysis.

### Ethic approval and data availability

The collection of clinical data and tumor samples were approved by the institutional review board of West China Hospital (No. 2018.569) following the 1964 Helsinki declaration and its later amendments. Besides, every patient signed written consent for collecting and using tumor tissue and clinical information. All the tumor tissue sequencing data from West China Hospital were available at the Genome Sequence Archive for Humans with accession code: HRA002839 (https://ngdc.cncb.ac.cn/gsa-human/s/XRStoK4w).

## Results

### Definition of essential arginine metabolism-related genes and construction of the arginine metabolism-related genes risk signature

**FIGURE 1:**
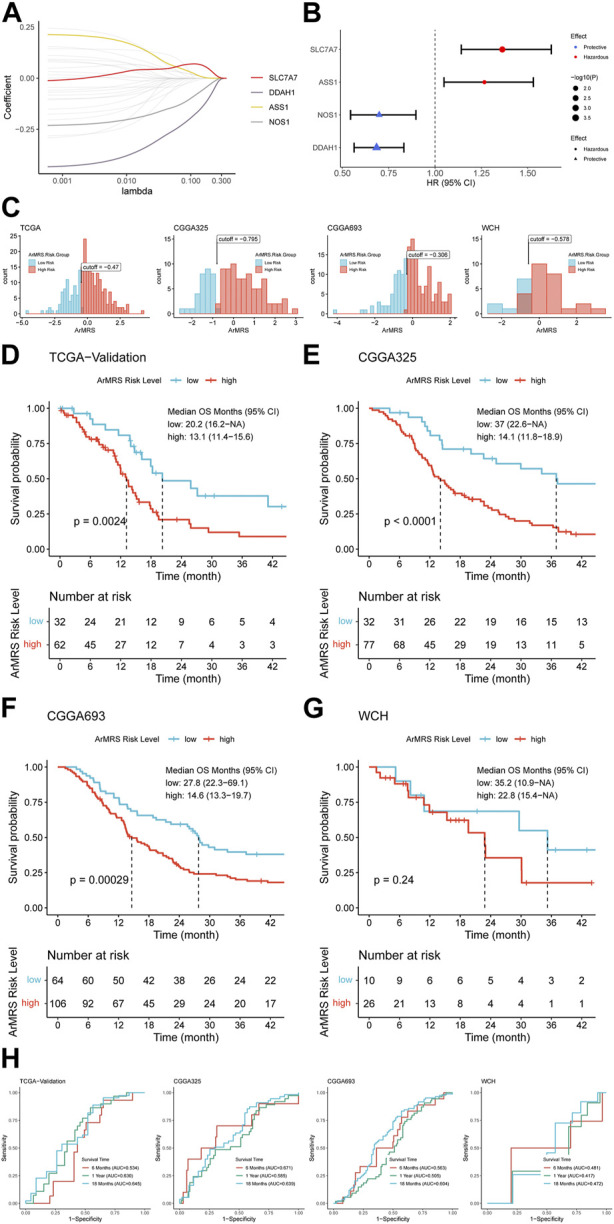
Construction of ArMRS and its efficacy on prediction of glioblastoma patients’ survival **(A)**. Average of coefficients of 4 essential ArMGs in the LASSO Cox regression at each lambda value **(B)**. The effect of every essential ArMG on the prognosis of glioblastoma **(C)**. Optima cutoff values of ArMRS in all four cohorts **(D)**. K-M curve of ArMRS risk groups in TCGA validation cohort, **(E)** CGGA325 cohort, **(F)** CGGA693 cohort, and **(G)** WCH cohort **(H)**. ROC curves and matched AUC of 6-, 12-, 18-month survival rate in all four cohorts.

First, we screened the 26 ArMGs with the LASSO Cox regression in the training set to determine essential genes for the construction of arginine metabolism-related genes risk signature (ArMRS). After that, four ArMGs, including SLC7A7, DDAH1, ASS1, and NOS1, were identified as essential ArMGs for the construction of ArMRS (Figure 1A). A calculation formula of ArMRS was also derived by fitting a final multivariate Cox regression model to the expression of the 4 essential ArMGs in the training set. The calculation formula of ArMRS was as following:

0.020*SLC7A7+0.007*ASS1−0.018*DDAH1−0.333*NOS1

Univariate analyses also demonstrated that SLC7A7 and ASS1 were hazardous prognostic factors for glioblastoma, while DDAH1 and NOS1 were proved as protective factors for glioblastoma (Figure 1B). Then we utilized the “surv_cutpoint” algorithm to determine the optimal ArMRS cut-off values for all these four cohorts. And based on these cut-off values, the patients of these four cohorts were allocated into ArMRS high-risk and low-risk groups (Figure 1C). Further survival analyses on the TCGA-validation cohort confirmed that the glioblastoma patients in the ArMRS high-risk group had remarkably poorer overall survival than in the ArMRS low-risk group (Figure 1D). This conclusion was also confirmed by survival analyses of CGGA325 and CGGA693 cohorts (Figures 1E, F). As to our own cohort, despite no statistical difference on the survival between two groups, there was also a trend that patients of high-risk group had poorer prognosis (Figure 1G). To evaluate the efficiency of ArMRS in predicting glioblastoma prognosis, we first performed ROC analyses to evaluate the performance of ArMRS alone in predicting glioblastoma patient survival at 6, 12, and 18 months. In the TCGA validation cohort, the AUCs of ArMRS at 6, 12, and 18 months were 0.534, 0.630, and 0.645, respectively (Figure 1H). Similar efficiencies were also observed in the other three validation cohorts (Figure 1H).

**FIGURE 2:**
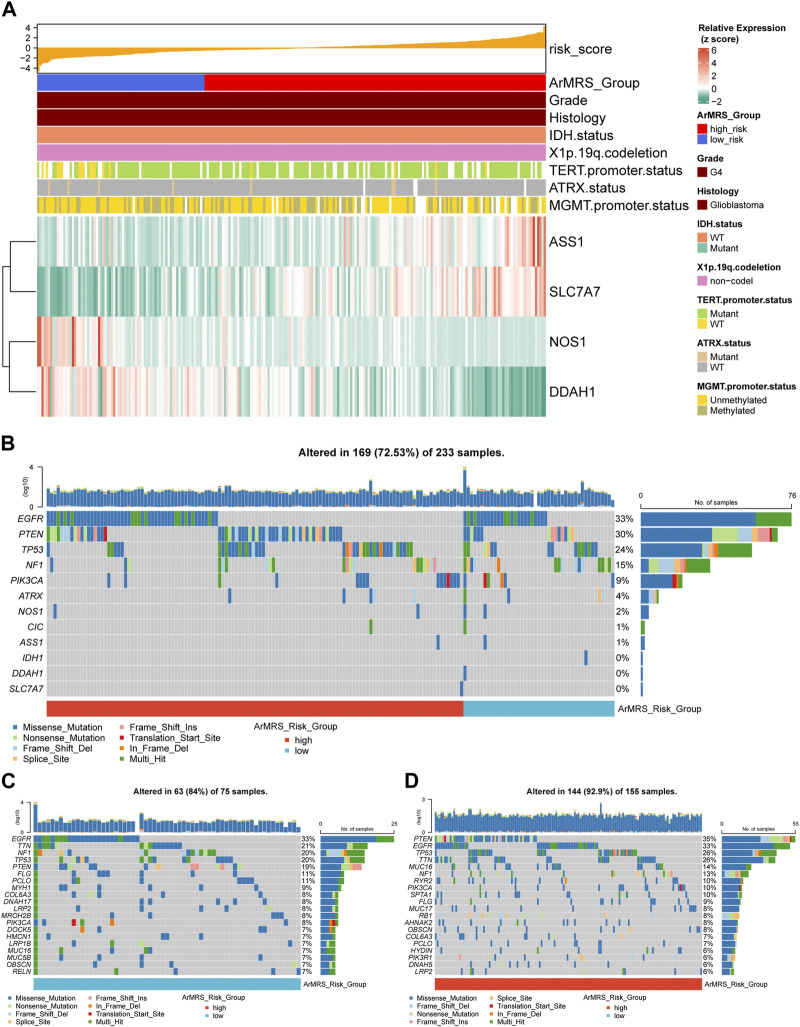
Expression level of four essential ArMGs and differences in gene mutations between ArMRS risk groups **(A)**. Expression level of four essential ArMGs and its relationship with clinicopathological features **(B)**. Gene mutations of four essential ArMGs and top eight frequently mutated genes in glioblastoma ordered by ArMRS risk groups **(C)**. Top 20 frequently mutated genes in ArMRS low-risk group **(D)**. Top 20 frequently mutated genes in ArMRS high-risk group.

**FIGURE 3:**
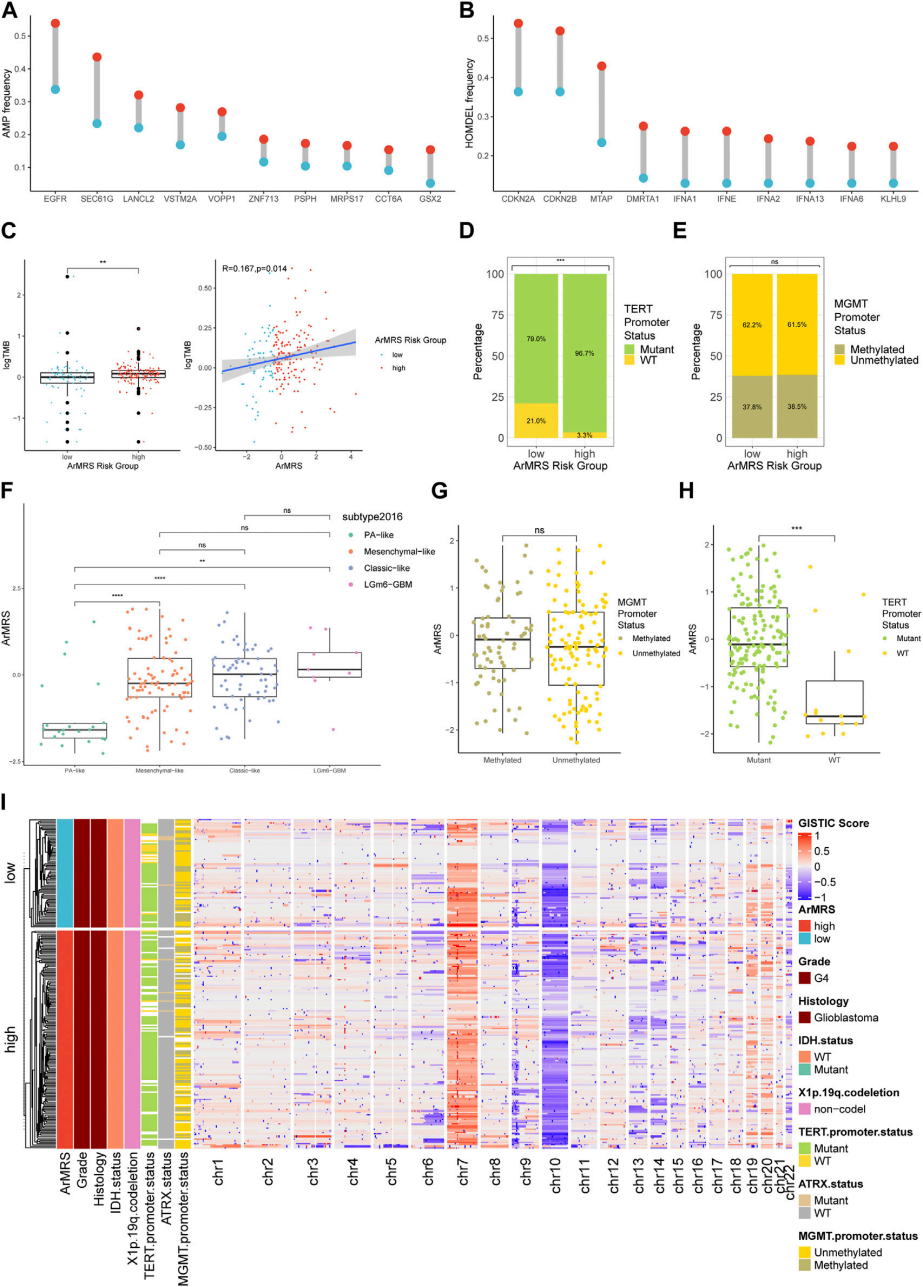
Differences in clinicopathological features and copy number variations between ArMRS risk groups **(A)**. Top 10 frequent amplification genes in ArMRS risk groups **(B)**. Top 10 frequently homozygously deleted genes in ArMRS risk groups **(C)**. Difference in tumor mutation burden between ArMRS risk groups and correlation between ArMRS and tumor mutation burden **(D)**. Difference in the incidences of TERT promoter mutation and **(E)** MGMT promoter methylation between ArMRS risk groups **(F)**. Difference in ArMRS among different subtypes of glioblastoma **(G)**. Difference in ArMRS between different MGMT promoter status, and **(H)** TERT promoter status **(I)**. Copy number variation and its relationship with clinicopathological features ordered by ArMRS risk groups. *p < 0.05; **p < 0.01; ***p < 0.001; ****p < 0.0001.

We also aligned a heatmap in the order of ArMRS that integrated the expression levels of these four essential ArMGs and clinicopathological characteristics, including TERT promoter status and MGMT promoter status (Figure 2A). As for the analyses of gene mutations, these four essential ArMGs rarely mutated in glioblastoma (Figure 2B), which excluded aberrant expression caused by gene mutations. The EGFR and TTN mutations were the most frequent mutations in glioblastoma of ArMRS low-risk group (Figure 2C). And PTEN and EGFR were the most frequent in ArMRS high-risk group (Figure 2D). Other mutations that ranked in top 20 most frequent for ArMRS low- and high-risk group were also listed. Furthermore, the incidence of EGFR amplification and CDKN2A/B homozygous-deletion were also significant higher in ArMRS high-risk group compared to low-risk group (Figures 3A, B). Moreover, the tumor mutation burden (TMB) analysis between ArMRS high- and low-risk groups revealed a significantly higher TMB in ArMRS high-risk group compared to low-risk group (Figure 3C). The analyses of clinicopathological features also showed that ArMRS high-risk group had a higher incidence of TERT promoter mutation (Figure 3D), but no differences in MGMT promoter status (Figure 3E). Besides, the pilocytic astrocytoma-like (PA-like) glioblastomas demonstrated significantly lower ArMRS than other three glioblastoma molecular subtypes (Figure 3F). There was also no difference in ArMRS between glioblastomas with methylated and unmethylated MGMT promoter (Figure 3G). But the glioblastomas with TERT promoter mutation had a significantly higher ArMRS than TERT promoter wild-type glioblastomas (Figure 3H), which suggested that the TERT promoter mutation may be associated with arginine metabolism. Additionally, the analysis of CNVs demonstrated that gain of chromosome 7 and loss of chromosome 10 occurred frequently both in ArMRS high- and low-risk groups (Figure 3I).

### Functional enrichment analyses based on ArMRS risk groups

**FIGURE 4:**
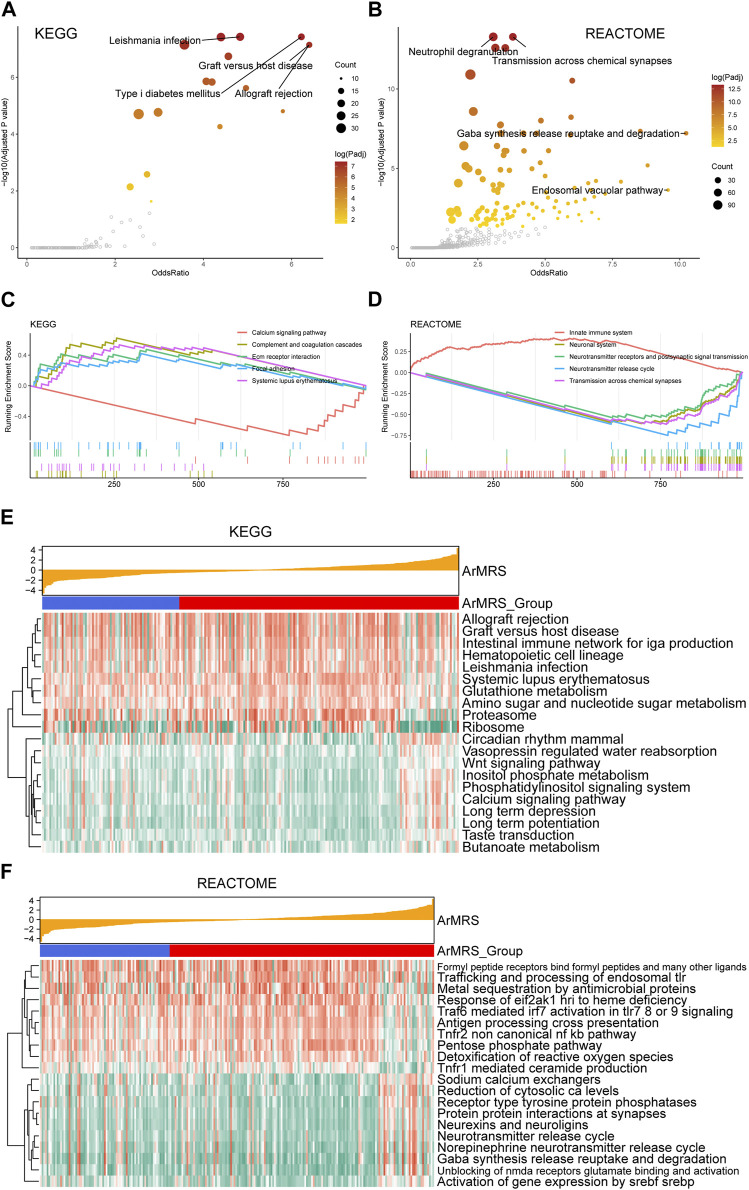
Functional enrichment analyses between ArMRS risk groups **(A)**. Pathways with high odds ratio and confidence in the KEGG gene sets **(B)**. Pathways with high odds ratio and confidence in the REACTOME gene sets **(C)**. The top five pathways with the highest normalized enrichment score in the KEGG gene sets between ArMRS risk groups **(D)**. The top five pathways with the highest normalized enrichment score in the HALLMARKS gene sets between two ArMRS risk groups **(E)**. Top 20 differentially expressed KEGG gene sets **(F)**. Top 20 differentially expressed REACTOME gene sets.

To evaluate the pathway alterations in different ArMRS risk groups, we conducted a series of functional enrichment analyses. The over-representation analysis illustrated the pathway alterations with high odds ratio and significance in the KEGG dataset, including allograft rejection and graft versus host disease (Figure 4A). The pathway alterations with high odds ratio and confidence in the REACTOME dataset were also illustrated (Figure 4B). Furthermore, extracellular matrix receptor interaction (normalized enrichment score (NES) = 2.285, adjusted *p*-value = 0.003) and the focal adhesion (NES = 2.261, adjusted *p*-value = 0.003) were ranked among the top five of the KEGG gene sets in the differentially expressed genes (DEGs) between ArMRS high- and low-risk groups (Figure 4C). Innate immune system (NES = 3.196, adjusted *p*-value < 0.001) and neurotransmitter release cycle (NES = −3.569, adjusted *p*-value < 0.001) were ranked in the top five of the REACTOME gene sets (Figure 4D). Finally, the GSVA result demonstrating the top 20 differentially expressed pathways in KEGG and REACTOME gene sets were illustrated through heatmaps (Figures 4E, F).

### Prediction of glioblastoma prognosis with ArMRS-Based nomograms

**FIGURE 5:**
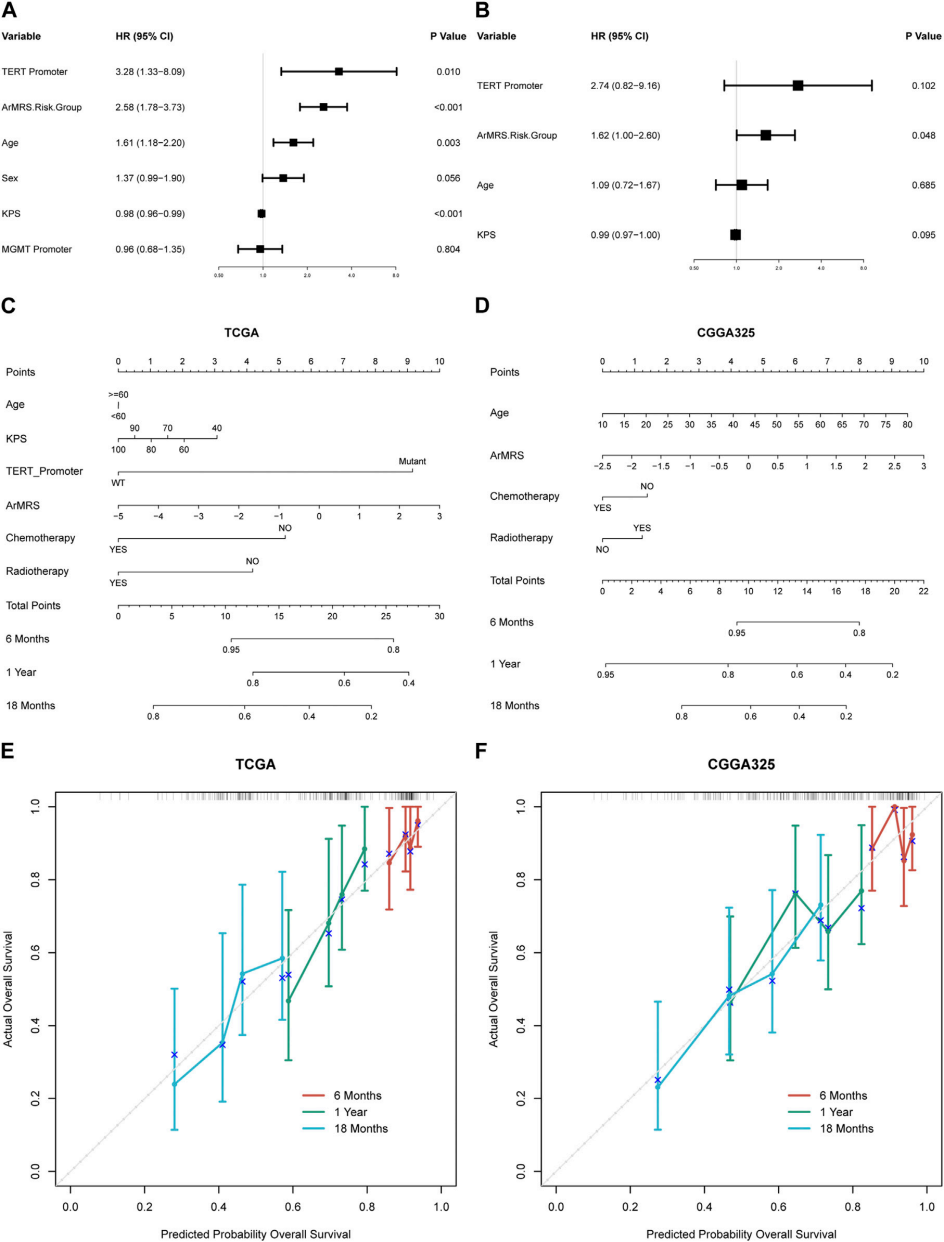
Prognostic value of ArMRS and construction of ArMRS-based nomograms **(A)**. Univariate and **(B)** Multivariate Cox regression analysis of potential prognostic factors in overall survival of glioblastoma. Nomogram of 6-, 12-, and 18-month survival of glioblastoma patients based on **(C)** TCGA cohort, **(D)** CGGA325 cohort. Calibration plots of the nomogram based on **(E)** TCGA cohort and **(F)** CGGA325 cohort.

To construct nomograms for the prediction of glioblastoma patients’ prognosis, we first conducted univariate followed by multivariate Cox analyses to determine potential independent prognostic factors. Result demonstrated that the age, ArMRS, and TERT promoter status were significant univariate prognostic factor (Figure 5A). Subsequently, these factors were enrolled in multivariate Cox regression analysis and the result revealed that ArMRS were independent prognostic factors in glioblastoma (Figure 5B). Eventually, we combined the potential prognostic patient factors as determined in the univariate analysis along with the adjuvant therapies to construct a nomogram for personalized survival prediction in the TCGA cohort (Figure 5C). Furthermore, the same process was also conducted to construct a nomogram for individualized survival prediction in CGGA325 cohort (Figure 5D). The corrected C-index of this nomogram based on TCGA cohort was 0.574. These two nomograms’ performance in predicting the prognosis of glioblastoma patients was validated by the 6-, 12-, and 18-month calibration curves (Figures 5E, F).

### Correlation of ArMRS with immune cells and immune microenvironment

**FIGURE 6:**
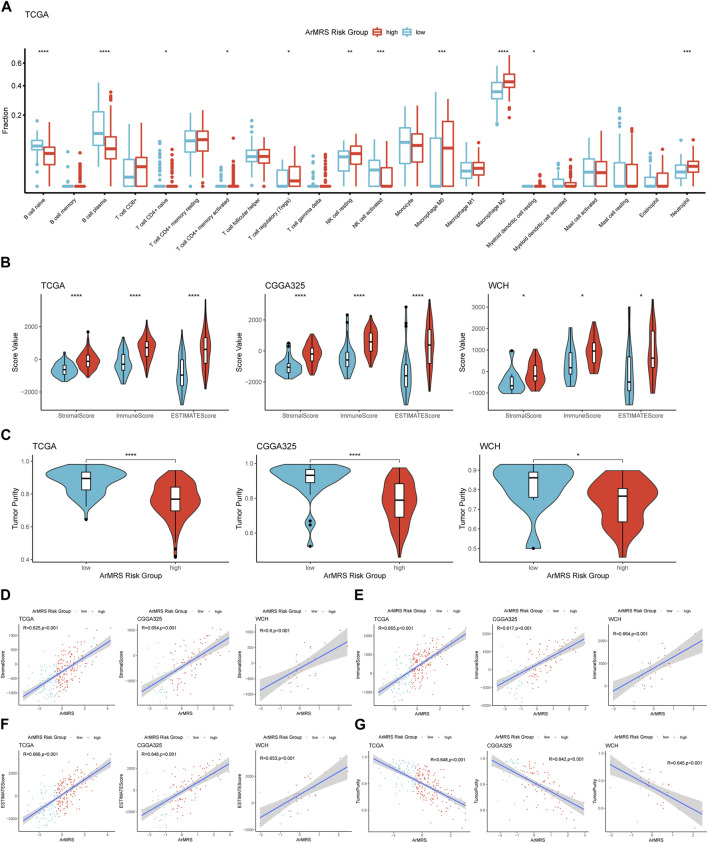
Comprehensive analyses on differences in immunological features between ArMRS risk groups **(A)**. Differences in the estimated infiltration fraction of 22 types of immune cells between glioblastomas of different ArMRS risk groups **(B)**. Differences in the stromal, immune, and ESTIMATE scores between ArMRS risk groups in TCGA, CGGA325, and WCH cohorts **(C)**. Differences in tumor purity between ArMRS risk groups in TCGA, CGGA325, and WCH cohorts **(D)**. Analyses of correlations of ArMRS with the **(D)** stromal score, **(E)** immune score, **(F)** ESTIMATE score, and **(G)** tumor purity in TCGA, CGGA325, and WCH cohorts. *p < 0.05; **p < 0.01; ***p < 0.001; ****p < 0.0001.

Finally, to elucidate the correlation between ArMRS and the immune landscape of glioblastomas, we conducted comprehensive analyses to evaluate the relationship between ArMRS and multiple immunity-related indexes. Firstly, we used the CIBERSORTx algorithm to compute the infiltration fractions of 22 types of immune cells in the tumor microenvironment. The results demonstrated that in the tumor microenvironment of ArMRS high-risk glioblastomas, there are more M2 macrophages and neutrophils and fewer plasma cells (Figure 6A), indicating different immune cell infiltration models between ArMRS high- and low-risk glioblastomas. Furthermore, we calculated the immune-related scores and tumor purity using the ESTIMATE algorithm. The results revealed that glioblastomas of ArMRS high-risk groups had remarkably higher stromal score, immune score, and ESTIMATE score compared to ArMRS low-risk groups in TCGA, CGGA325, and WCH cohorts (Figure 6B), suggesting more complex tumor microenvironment in glioblastomas with higher ArMRS. Subsequently, the analyses of tumor purity also confirmed that glioblastomas of the ArMRS high-risk groups had significantly lower tumor purity compared to low-risk groups (Figure 6C), in line with the results of immune-related scores. Additionally, correlation analyses also confirmed that the stromal score, immune score, and ESTIMATE score were positively correlated with the value of ArMRS in these cohorts (Figures 6D, F). The tumor purity was negatively correlated with the value of ArMRS in these cohorts (Figure 6G).

**FIGURE 7:**
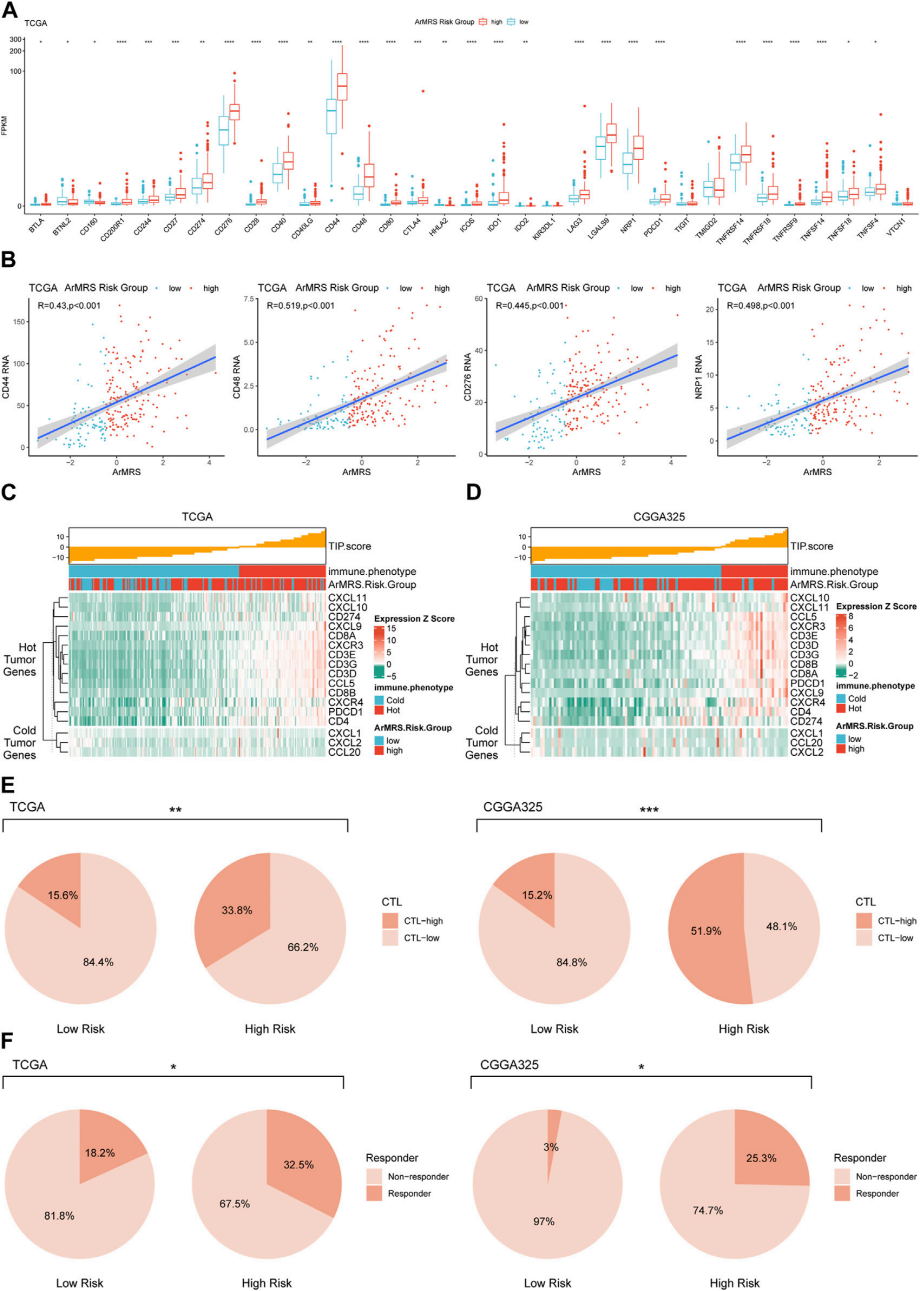
Differences in potential respond to immunotherapy in glioblastomas of different ArMRS risk groups **(A)**. Differences in the expression level of 33 immunotherapy-related genes between ArMRS risk groups in TCGA cohort **(B)**. Analyses of correlations between ArMRS and the expression levels of CD44, CD48, CD276, and NRP1 in TCGA cohort **(C)**. Analysis of tumor immunological phenotype (TIP) score and related gene expression levels ordered by ArMRS in TCGA cohort **(D)**. Analysis of TIP score and related gene expression levels ordered by ArMRS in CGGA325 cohort **(E)**. Difference in proportion of patients with high cyto-toxic T lymphocytes infiltration between ArMRS risk groups in TCGA and CGGA325 cohort **(F)**. Difference in proportion of patients with predictive response to immune checkpoint inhibitors between ArMRS risk groups in TCGA and CGGA325 cohort.

Moreover, to investigate the relationship between ArMRS and response to immunotherapy, we analyzed the correlation between multiple immunotherapy-related markers and ArMRS. The results revealed that glioblastomas of ArMRS high-risk group had significantly higher expression levels of CD44, CD48, CD276 (B7-H3), and PD1 (PDCD1) compared to low-risk group in the TCGA cohort (Figure 7A), suggesting higher ArMRS may indicate more abundant expression of immunotherapy targets. Additionally, the ArMRS showed significant positive correlation with expression of CD44, CD48, CD276, and NRP1, which is a inhibitory immune checkpoint involved in M2 polarization of microglia and TGF-β release from regulatory T cells (Figure 7B) (Roy et al., 2017). Besides, we also computed the TIP score to identify the relationship between immunological phenotype and ArMRS in glioblastoma. The result demonstrated that the glioblastomas of ArMRS high-risk group in TCGA cohort would highly express genes associated with relatively ‘hot’ tumor immunological phenotype (Figure 7C). This observation was also confirmed in the CGGA325 cohort (Figure 7D). Additionally, the analysis of cytotoxic T cells (CTLs) revealed that the glioblastomas of ArMRS high-risk group harbored more CTLs infiltration compared to the low-risk group in TCGA and CGGA325 cohort (Figure 7E). Immune checkpoint inhibitor response prediction with the TIDE suite revealed that the glioblastoma patients of ArMRS high-risk group were more likely to benefit from therapy of immune checkpoint inhibitors in the TCGA and CGGA325 cohorts (Figure 7F).

## Discussion

Malignant tumors of central nervous system cause at least 200 thousand death worldwide every year (Siegel et al., 2021). As the most common malignant tumor of central nervous system, glioblastoma is responsible for more than a half of these deaths. Despite abundant attempts worldwide try to search novel potential therapy for glioblastoma, there is still hardly any satisfactory breakthrough d in about recent 20 years. After thorough treatment regime, which contains surgery, radiotherapy, chemotherapy with temozolomide, and even tumor treating fields, the median overall survival of glioblastoma patients was still less than 2 years (Stupp et al., 2005; Stupp et al., 2017). Immunotherapy, as an extremely attractive breakthrough in cancer treatment, have succeeded to improve the length and quality of patients in many cancers (Eggermont et al., 2018; Gandhi et al., 2018; Choueiri et al., 2021; Cortes et al., 2022; Luke et al., 2022). Spontaneously, researchers have high expectations on immunotherapy to break through the dilemma of glioblastoma treatment. However, studies about immunotherapy in glioblastoma have faced unprecedented challenges, and almost all these attempts failed to improve overall survival (Weller et al., 2017; Wakabayashi et al., 2018; Reardon et al., 2020; Lim et al., 2022; Omuro et al., 2022). There are many potential reasons that impede immunotherapy to work for glioblastoma treatment. One of these reasons is that brain has a totally distinctive immune landscape compared to other organs. Endothelial cells that form blood vessels in brain are unique compared to other tissues, and compose a unique structure, the blood-brain barrier (BBB), which functions to prevent most peripheral immune cells from entering brain and subsequently form an immunological quiescent environment (Obermeier et al., 2013). Therefore, in a long period, brain is considered as an immune privilege site, resulting in failures of immunotherapy. However, recent studies have proved that even with the existence of BBB, brain can exchange immune cells with circulation sites through a novel lymphatic pathway (Louveau et al., 2015). Through this transport method, the peripheral T and B lymphocytes can be primed and then infiltrate to brain and deliver immune effects in brain (Lim et al., 2018). These studies inspire us that brain is not a forbidden zone for immunotherapy. With further and better understand of immune landscape of glioblastoma, immunotherapy is still with great potential to improve length and quality of glioblastoma patients’ life.

The correlation between reshaped metabolic model of tumors and immunological landscapes has attracted surging attention (Xia et al., 2021). Our previous studies also suggested that the metabolism of specific compounds of cancer cell, including purine, tryptophan, serine, and glycine was tightly related to immunological features of glioma (Chen et al., 2022a; Chen et al., 2022b; Zhang et al., 2022). However, we also found that any of these metabolism pathways can totally explain the unique metabolism characteristics of glioma. There must be many other metabolism pathways that influenced and reshaped the immunological features of tumor microenvironment of glioma. Many evidence have indicated that arginine metabolism plays an essential role in many physiological processes of cells (Delage et al., 2010). And the arginine metabolism in cancers is totally reshaped because of the lack of ASS1, a key enzyme during the production of arginine (Bronte and Zanovello, 2005). Therefore, cancer cells must obtain arginine from tumor microenvironment to meet their own demands for arginine, which would lead to arginine deficiency in the tumor microenvironment. It has been proved that arginine deficiency would increase the expression of PD-1 (Mussai et al., 2019), and decrease the proliferation rate of chimeric antigen receptor T cells (CAR-T cells) (Fultang et al., 2020). Therapeutic attempts have demonstrated that supplementation of arginine might potentiate the response to immune checkpoint inhibitors in tumors (He et al., 2017; Canale et al., 2021). Arginine could also evoke metabolic adaption in brain metastases and enhances therapeutic effects of radiation (Marullo et al., 2021).

After filtering ArMGs, four ArMGs were determined as essential genes for glioblastoma patients’ prognosis. For example, Solute Carrier Family 7 Member 7 (SLC7A7), which encodes the light chain of a cationic amino acid transport system (He et al., 2009), could function to transport cationic amino acids such as arginine and lysine across cell membrane (Kanai et al., 2000). It has been proved that glioblastoma overexpressed SLC7A7 compared to normal brain tissue, and overexpression of SLC7A7 was correlated with poor prognosis in glioblastoma (Fan et al., 2013). Overexpressed SLC7A7 could accelerate the velocity that cancer cells obtained arginine from tumor microenvironment, and then exacerbate the arginine deficiency in tumor microenvironment. Besides, ASS1, as a key enzyme in the production of arginine, was also identified as an essential gene. Higher ASS1 expression level was correlated with poorer prognosis, indicating that the production of arginine could relief the arginine deficiency in tumor but may not be able to improve prognosis. Because although extracellular arginine can function to enhance the anti-tumor effects of immune cells, the tumor cells also could utilize the high concentration of extracellular arginine to replenish intracellular arginine pool. Deprivation of arginine is cytotoxic to glioblastoma cells which lack ASS1(Khoury et al., 2015).

In the last part of this study, we focused on the relationship between arginine metabolism and immunological features of glioblastoma. To achieve this goal, we utilized multiple algorithms from independent studies to comprehensively analyzed the differences in immune microenvironment between ArMRS high- and low-risk groups. First, we utilized the CIBERSORTx algorithm, which aimed to analyze immune cells infiltration, to analyze the differences in the infiltration fractions of 22 types of immune cells between ArMRS risk groups. The results revealed that the infiltration of plasma cells and M2 macrophages were strongly correlated with ArMRS. For instance, M2 macrophages in the tumor microenvironment were generally believed as pro-tumor subtype of macrophages, can promote tumor proliferation and suppress local anti-tumor immunity (Noy and Pollard, 2014). In our study, we found that the infiltration of M2 macrophages in ArMRS high-risk group was enormously greater compared to low-risk group. This finding suggested that higher ArMRS value, which represented for more arginine deficiency, was a marker for more M2 macrophage infiltration and suppressed anti-tumor immunity. Moreover, the ESTIMATE analyses demonstrated that the complexity of tumor microenvironment of glioblastoma was strongly positively correlated with the ArMRS, indicating that more arginine deficiency might contribute to reconstructing a more complex tumor microenvironment in glioblastoma. The expression levels of multiple targets for immunotherapy, for example, PD-1, were also tightly positively correlated with the ArMRS. The high expression level of immunotherapy targets, including PD-1 and PD-L1, was considered as a signal for better response to corresponding immunotherapy (Reck et al., 2016; Bajorin et al., 2021). Therefore, the correlation between ArMRS and expression of PD-1, which was introduced in this study, might endorse the potential ability of ArMRS to predict the response to immunotherapy. Higher ArMRS was also correlated with more potential responders to ICIs and more expression of ‘hot tumor’ features, which can validate the previous results. All these results from multiple algorithms on immunological features indicated that high ArMRS would predict worse prognosis and more suppression of anti-tumor immunity. But it also represented for more expression of multiple immunotherapy targets, which endorsed the potential ability of ArMRS to direct the application of immunotherapy in glioblastoma. Additionally, the combined application of ArMRS and prognostic scores of our previous studies may provide more comprehensive evaluation tools for each patient. For example, the ArMRS could guide the application of novel therapies targeted on arginine metabolism. If a novel therapy targeted on another metabolism pathway, to use the prognostic score that based on that metabolism pathway may be better. And comprehensive application of these prognostic scores may provide more choice and accuracy than every single one.

Despite multiple analyses can endorse same results and function as a validation for each other in our current study, there were still multiple limitations. First, due to the four patent cohorts were from different databases, the protocol of sequencing and data preprocessing differed among these four independent cohorts. Second, these findings of our current study were totally based on mRNA sequencing data and consequently required further basic experiment validation and more convincing evidence. Next, like our previous studies, we focused on one metabolism pathway, the arginine metabolism, in this study. This result in that our study can only depict the relationship between metabolism and immunological features from one perspective. Together with our previous studies, we are trying to depict this relationship as detailed as possible. But there must be many other metabolism pathways that impacted on the formation of immunological features of glioblastoma, which calls for more comprehensive research. Finally, the mechanism of how arginine metabolism impacted the immunological feature of glioblastoma remained blurred and demands further research.

## Conclusion

In conclusion, we constructed a novel arginine metabolism evaluation score system based on four essential arginine metabolism-related genes, ArMRS, which showed for a strong ability to predict prognosis of IDH-wildtype glioblastoma patients. Besides, higher ArMRS, which represents for more arginine deficiency, was correlated with more immune infiltration, more immunosuppression, and more expression of targets for immunotherapy, which endorsed the usages of ArMRS in directing immunotherapy in glioblastoma.

## Data Availability

According to national legislation/guidelines, specifically the Administrative Regulations of the People's Republic of China on Human Genetic Resources (http://www.gov.cn/zhengce/content/2019-06/10/content_5398829.htm, http://english.www.gov.cn/policies/latest_releases/2019/06/10/content_281476708945462.htm), no additional raw data is available at this time. Data of this project can be accessed after an approval application to the National Genomics Data Centre (NGDC, https://ngdc.cncb.ac.cn/). Please refer to https://ngdc.cncb.ac.cn/ for detailed application guidance. The accession code HRA002839 should be included in the application.
